# Carbamazepine Induced Stevens-Johnson Syndrome That Developed into Toxic Epidermal Necrolysis: Review of the Literature

**DOI:** 10.1155/2022/6128688

**Published:** 2022-05-06

**Authors:** Yousef S. Abuzneid, Hussam I. A. Alzeerelhouseini, Duha Rabi, Ihab Hilail, Hatem Rjoob, Abdelrahman Rabee, Naser Amro, Qutaiba Qafisheh, Mohammad Kharraz

**Affiliations:** ^1^Al-Quds University Faculty of Medicine, Jerusalem, State of Palestine; ^2^An-Najah National University Hospital, Nablus, State of Palestine

## Abstract

**Background:**

Stevens-Johnson syndrome and toxic epidermal necrolysis are both skin diseases believed to be following the pattern of a type IV hypersensitivity mechanism, which can be triggered by infectious agents or administration of a variety of drugs as part of the spectrum of severe cutaneous adverse reactions (SCARs). Fever and blisters, that peel forming painful raw areas, are early symptoms of this condition, and complications such as dehydration, sepsis, pneumonia, and multiple organ failure are typically seen during the course of the disease. *Case Presentation*. We present a case of a 23-year-old female patient referred to our hospital after taking carbamazepine and developing high-grade fever and ulcers that appeared initially in her mouth and face but then progressed despite treatment, extending all over her body and involving about 90% of her BSA.

**Conclusion:**

The use of IVIG and plasmapheresis was a good management for our case, helping in our patient's well-being and recovery. Even if there is no stipulated guideline treatment for cases of SJS and TEN, we think that further investigations about IVIG and plasmapheresis should be investigated as a possible way to treat both conditions.

## 1. Introduction

Stevens-Johnson syndrome (SJS) and toxic epidermal necrolysis (TEN) are both rare skin reactions that are often triggered by particular medications and can be life-threatening. Antiepileptic drugs (AEDs), nonsteroidal anti-inflammatory drugs (NSAIDs), and certain antibiotics have been shown to trigger these reactions [1].

At the beginning, SJS and TEN were addressed as different conditions, but now they are considered as two forms of the same skin disease. SJS is defined as detachment of less than 10% of the total body surface area (BSA), while TEN is the detachment of greater than 30% of the total BSA [2].

Both conditions are characterized by full-thickness epidermal necrosis and severe mucous membrane inflammation leading to mucocutaneous sloughing and denudation [3].

It affects all age groups, affecting more those individuals that have HIV, autoimmune diseases, immunocompromised patients, and those who have an underlying malignancy. Also, it is thought to be an immune mediated drug reaction with higher rates in certain population (for example, carbamazepine associated SJS/TEN has been linked to Han Chinese and other Asian groups due to HLA-B^*∗*^1502) [4].

The incidence of these reactions is estimated to be 1.5–8.3 cases per 1000000 person-year. The mortality from SJS is approximately 5%, whereas the mortality from TEN is approximately 30%. There are studies showing that even after one year of hospitalization, the mortality risk for those patients who suffer from SJS/TEN continues.

This condition appears to be more lethal in the early phase of the disease. Older age and the presence of comorbidities are two significant risk factors that can increase the mortality rate [1]. It has been reported that patients who survive SJS/TEN are at high risk of developing long-term complications involving the skin, eyes, mucosa, and respiratory, renal, and/or hepatic systems [1].

## 2. Case Presentation

We present a 23-year-old female patient from Gaza, with a history of medicated major depressive disorder. She was in her usual state of health until she started developing multiple painful ulcers over her face and oral cavity after 10 days of the addition of carbamazepine to her treatment regimen for her headaches and anxiety disorder. She sought immediate medical advice and was diagnosed with Stevens-Johnson syndrome and was put on hydrocortisone (100 mg × 2) with no improvement.

The rash rapidly progressed over her entire body, and she developed high-grade fever (41°C). Thus, antibiotics were added, and she was referred to our hospital.

**Figure 1:**
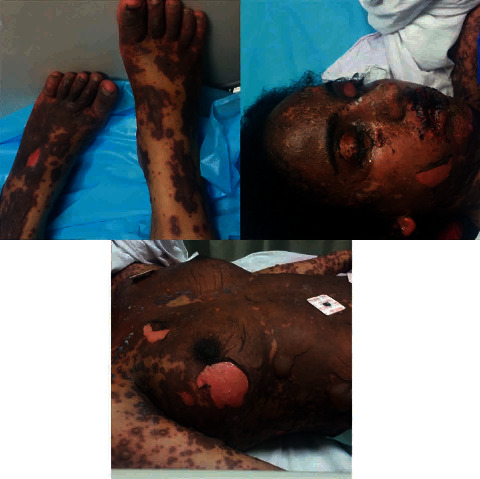
Diffuse skin desquamation resembling burns covering almost 90% of the patient's body.

When she arrived, the patient was confused and could not be aroused. She had ulcerations resembling burns covering her entire body with diffuse skin desquamation, sloughed skin, high-grade fever, and severe dehydration (Figure 1). Her vital signs showed a blood pressure of 80/40 and heart rate of 130 beats/min. WBC was 24 × 10^9^/L and procalcitonin was 4 ng/mL, making septic shock clearer. The patient had more than 10% of body surface area involved, tachycardia, a serum glucose of 74 mg/dL (4.1 mmol/L), a serum urea of 39 mmol/L, and a serum bicarbonate of 12 mmol/L; so, her SCORTEN score (severity-of-illness score for toxic epidermal necrolysis) was calculated to be 4.

Furthermore, a central line was inserted in ER carefully because of extensive skin desquamation, and an IV normal saline and broad-spectrum antibiotics were started which included tigecycline, colistin, and fluconazole. Skin culture was done, showing heavy growth of Gram-negative bacteria bacilli that was later found to be a multidrug-resistant *Acinetobacter baumannii* strain. Thus, she remained on the same medication regimen.

Due to the extensive desquamation of almost 90% of her body, the patient's case was diagnosed as toxic epidermal necrolysis, which is known to have a high mortality rate. Our patient's situation was especially progressive and severe, necessitating a DNR order discussion with her family.

Pulse steroids with Solu-Medrol (methylprednisolone sodium succinate that was given 1 g × 1) were also initiated, and medical consultation was sought in order to start plasma exchange treatment and plastic surgery.

After 6 sessions of plasmapheresis in addition to IVIG, we noticed that the patient condition started to improve, the fever disappeared, and the desquamation of the skin started to heal.

Afterwards, an ophthalmologist consultation was required because the patient had developed a complication of closure of the left eye due to the necrosis of the periorbital skin which needed to be opened surgically.

The patient was discharged after 28 days and then had follow-up appointments by regular call phones (since people from Gaza need a special permit to come to the West Bank). She was advised not to take anticonvulsants because of their well-established side effects (including SJS and TEN). She was also recommended to take SNRIs (serotonin and norepinephrine reuptake inhibitors). After six months, she presented to our hospital in a good condition with excellent performance.

## 3. Discussion

The pathophysiology of the disease is not fully understood, but it is believed to be a delayed hypersensitivity reaction mediated by Th1 cells which is the cause of keratinocyte apoptosis by Fas (CD95) and FasL ligand (CD95L) interaction and the release of perforin and granzyme B pathways [5].

SJS and TEN have many causes, but drugs are the most common. Antiepileptics (lamotrigine, carbamazepine, phenytoin, and phenobarbital), allopurinol, oxicam, nonsteroidal anti-inflammatory drugs, and sulfa drugs are the most implicated. Other causes such as infection and malignancy were also reported, and the risk of developing the disease increases in immunocompromised and HIV-infected patients [6].

There is a strong association between some HLA alleles and drug-induced SJS/TEN. The most significant is HLA-B^*∗*^15 : 02 with carbamazepine-induced SJS among the Asian population. Genetic screening for HLA-B^*∗*^15 : 02 can be performed prior to the prescription of carbamazepine, especially in Southeast Asia, where the prevalence of this allele is high [3]. Unfortunately, in our case, we cannot confirm that the allele is the cause for TEN as a genetic test was not performed.

The clinical presentation of SJS and TEN mainly consists of three phases [2]:

1. Prodromal phase: present 2-3 days before the acute phase and characterized by fever (100% of cases); flu-like symptoms such as cough, malaise, myalgia, arthralgia; and anorexia. Other symptoms may also present in this phase, such as conjunctivitis (32% of cases) and pharyngitis (25% of cases).
2. Acute phase: characterized by tender, painful macular eruptions with nonblanching “targetoid lesions,” which classically develop symmetrically on the face and trunk and spread to the extremities.
3. Lesions may become bullous and rupture, leaving denuded skin susceptible to a secondary infection. Mucosal involvement can also occur, involving pharyngeal, tracheal, bronchial, gastrointestinal, and vaginal regions.
4. Reepithelialization period: may take 3–6 weeks and secondary infection can increase the risk of scar formation.

Patients with TEN can develop multiorgan involvement resulting in renal, gastrointestinal, respiratory, and cardiovascular damages. However, sepsis from a secondary infection is the most common cause of death [6].

There are no specific laboratory tests (other than biopsy) that can definitively establish the diagnosis of SJS and TEN. Therefore, diagnosis is clinical, with emphasis on previous drug usage. CBC may reveal normal or nonspecific leukocytosis, but marked WBC elevation may indicate superimposed bacterial infection. So skin, urine, and blood cultures have been advocated because the incidence of serious bacterial bloodstream infections and sepsis significantly contribute to morbidity and mortality [5].

On skin biopsy, the disease is characterized by widespread necrotic keratinocytes, lymphocytic infiltration, and full-thickness epidermal necrosis with sloughing from the dermal-epidermal junction which is the cause of positive Nikolsky's sign (separation of epidermis at the subbasilar plane with gentle lateral pressure) [6]. Immunohistochemical studies revealed that CD8+ T lymphocytes predominate in the epidermis, whereas CD4+ T lymphocytes predominate in the dermis [2].

Management of patients with SJS or TEN mainly requires immediate withdrawal of the drug, which are the most important actions affecting significantly morbidity and mortality. Then, supportive and symptomatic treatment should be performed in intensive care units or burn centers, which include body temperature control, hydration, and electrolyte replacement, special attention to the airways, preventing secondary infection, pain control, maintenance of venous access distant from the affected areas, early oral nutrition or parenteral nutrition (if necessary), and anticoagulation [5].

Skin lesions are treated in the same manner of extensive burns treatment. Topical antiseptics can be used, but silver sulfadiazine should be avoided due to the association between SJS and sulfonamides [7].

Prophylactic antibiotic therapy is not recommended as it can increase microbial resistance. Some antibiotics can also trigger SJS/TEN, so their use should only be in cases of proven infection, a positive bacterial culture, the presence of high-grade fever, or significant leukocytosis [8]. In our case, the presence of high-grade fever increases the suspicion of infection, so a prophylactic antibiotic was given till the culture revealed multidrug-resistant *Acinetobacter baumannii* infection.

Active interventions specific to SJS and TEN are controversial. The low prevalence of the disease and its high mortality rate make it difficult to perform randomized clinical trials on SJS/TEN patients. Some reviews concluded that steroids do not shorten the duration of disease and may also increase the risk of sepsis and delay wound healing. Many authors do not recommend the routine use of systemic steroids in the treatment of SJS/TEN; however, some centers advocate an early pulse (first 48 hours) [7, 8].

Viard et al., in 1998, reported that commercial preparations of IVIG contained anti-Fas (anti-CD95) antibodies that blocked Fas-FasL interaction, thus intervening in disease the pathogenesis [9]. Successful treatment depends on the dose and its early use [10–12]. In our case, plasmapheresis and IVIG showed vast improvement in the patient's condition.

Mainly, the prognosis is significantly linked to early diagnosis with rapid identification of the causative drug and its discontinuation and appropriate treatment initiation. The prognosis does not seem to be affected by the type or the dose of the drug [5].

The SCORTEN score which was introduced in 2000 by Bastuji-Garin et al. [13] can be used to determine the prognosis and mortality rate in patients with SJS and TEN. Interestingly, one study published by some of the authors of the original SCORTEN paper found that the predictive value of the SCORTEN is most accurate when calculated on the 3rd day of hospitalization. Therefore, Guegan et al. [14] suggested that the SCORTEN score should be calculated not only on day 1 but also on day 3 of hospitalization [15]. Other factors associated with poor prognosis include late removal of the causative drug and delay in transfer of the patient to the burn unit [5].

## 4. Conclusion

In our case, treatment with plasmapheresis and intravenous immunoglobulin therapy were a successful management for the patient. Thus, we hope that our case will help other physicians in the treatment of toxic epidermal necrosis and Stevens-Johnson syndrome, since there is no stipulated and definitive treatment to both conditions, and they are both severe and life-threating diseases that need prompt management. Hence, we suggest more investigations regarding the management of these conditions hoping that in the future a specific treatment will be available.

## Data Availability

The data used to support this case report are from previously reported studies and datasets, which have been cited.
